# The Oropharyngeal Dysphagia Screening Test for Patients and Professionals: Validation in Cognitive Impairment and in Severe Mental Illness

**DOI:** 10.1007/s00455-024-10707-0

**Published:** 2024-06-13

**Authors:** Sara Mata, Blas Blánquez, Francisca Serrano

**Affiliations:** 1 The Mind, Brain and Behavior Research Center, University of Granada (CIMCYC-UGR), University of Granada, Granada, Spain; 2 Benito Menni Mental Health Care complex, SantBoi de Llobregat, Barcelona, Spain

**Keywords:** Oropharyngeal dysphagia, Screening, Dementia, Intellectual disability, Severe mental illness

## Abstract

Dysphagia is a symptom that appears with high prevalence in persons diagnosed with dementia, intellectual disability, or severe mental illness. Risk of aspiration pneumonia or even death is very high in these populations. However, screening for dysphagia risk in these patients is complicated by the fact that most of them suffer from cognitive impairments and behavioral manifestations that hinder the assessment process using the existing screening tests. The aim of this study was to validate the Oropharyngeal Dysphagia Screening Test for Patients and Professionals, in patients with cognitive impairment (dementia/intellectual disability) or with severe mental illness (schizophrenia and other psychotic disorders, bipolar disorder, or major depressive disorder). For this purpose, 148 institutionalized patients were evaluated by professionals responsible for their food intake. The Oropharyngeal Dysphagia Screening Test for Patients and Professionals was used to assess its validity in screening for oropharyngeal dysphagia in patients with cognitive impairments and in patients with severe mental illness. Also, the Eating Assessment Tool-10 and the Swallowing Disturbance Questionnaire were used for convergent reliability procedures. Four comparison groups were established: patients with cognitive impairment with and without oropharyngeal dysphagia, and patients with severe mental illness with and without oropharyngeal dysphagia. Results from the Oropharyngeal Dysphagia Screening Test for Patients and Professionals adequately distinguished between groups with and without dysphagia, in addition to presenting adequate levels of convergent validity and reliability. These results were obtained from other-reports (professionals responsible for patients’ food intake), using a simple, quickly applied test that does not require the use of food in patients with an altered cognitive state or with severe mental illness. With this study we expand the validity of the Oropharyngeal Dysphagia Screening Test for Patients and Professionals in populations with severe cognitive deficits and mental illness in which there is a great deficiency of oropharyngeal dysphagia screening instruments.

Dysphagia is a symptom of several diseases which have an affectation of the neural centers of deglutition, anatomical structures, or the orofacial musculature related to the swallowing process [1]. The incidence of dysphagia in the world is approximately of 8% of the population [2, 3]; the most prevalent type is oropharyngeal dysphagia (OD). OD has a high negative impact because it can potentially cause complications if left untreated, which can result in serious injury or even death [4–6], mainly due to aspiration pneumonia [7–9]. Additionally, a higher economical cost is associated with OD due to the increase in hospital stay and the cost of the patient care [10]. Finally, OD can also affect the patient’s quality of life and emotional state, as it can often be associated with depression or anxiety symptoms related with eating situations [10, 11]. To achieve an early diagnosis that makes it possible to apply treatment is therefore an objective for the healthcare community. However, identifying the risk of dysphagia is not always easy, due to the high number of patients who are at risk for presenting either an altered cognitive state or behavioral manifestations that interfere with the usual screening procedures.

It is estimated that OD prevalence falls between 84 and 93% in patients with dementia [12] and between 15 and 50% of patients with intellectual disability (ID) [13–15].

The high prevalence of dysphagia in persons with dementia is due to several factors. On one hand, there are impairments in attentional processes, in the state of consciousness, and in motor and sensory skills (sight/smell) [16]. These impairments hinder or prevent self-feeding, that is, connecting with the feeding situation and planning the process of selecting one’s food, handling it with the appropriate instruments and transporting it to the mouth [16]. On the other hand, the progression of dementia is associated with damage to brain structures that affect swallowing processes (tempoparietal, corticobulbar or frontotemporal lesions depending on the type of dementia) and that cause altered swallowing patterns: slower bolus transit, reduced hyolaryngeal movement, inadequate pharyngeal clearance, inadequate glottic closure, reduced esophageal sphincter opening, penetration/aspiration [16]. In the literature review by Foley et al. [17], the odds of death resulting from pneumonia were significantly increased for persons with dementia compared with those without dementia (OR = 2.22, 95% CI 1.44–3.42, *p* < 0.001). For persons with Alzheimer’s disease, the odds of death resulting from pneumonia were also significantly higher (OR = 1.70, 95% CI 1.44–3.42, *p* < 0.013).

In the case of intellectual disability (ID), the appearance of dysphagia is due to oral-motor dysfunction, neurological affectations, reflux, musculoskeletal deformities, and some anatomical abnormalities together with psychiatric and behavioural disorders [18]. An estimated 40% of adults with ID and dysphagia present repeated episodes of aspiration pneumonia, and this is the primary cause of death in patients with profound ID [15, 19]. Despite these figures, dysphagia in persons with ID is underdiagnosed. In their study with 176 institutionalized ID patients, Sheehanet al. [20] showed that only 19.3% had been assessed for risk of dysphagia. For their part, Bastiaanse et al. [21] showed that in 89.5% of the medical reports of persons with ID, swallowing disorders were not considered.

Less research has been conducted on the presence of dysphagia in severe mental illness (SMI) [22]. This term encompasses disorders such as schizophrenia, other psychotic disorders, bipolar disorder, and major depressive disorder, where patients show a severe impairment maintained for a prolonged period [22]. Nonetheless, it also proves to be highly present in these patients, as much as 32% in an institutionalized population [23]. The fundamental causes of dysphagia in patients with SMI relate to the use of psychotropic drugs [22–24], and to behavioral manifestations associated with SMI [24].

On the medication side, the use of antipsychotics has been considered responsible for symptoms related to extrapyramidal side effects (EPS) [22, 24]. For instance, drug-induced parkinsonism due to interference with dopamine receptors in nigrostriatal pathways [24]. This interference generates the bulbar symptoms characteristic of Parkinson’s disease and, therefore, difficulties in the phases of swallowing. This bulbar symptoms affects to the oral phase by poor tongue control and formation, poor bolus control and transit, poor closure of the posterior oral sphincter [24]. The bulbar symptoms also affects to the pharyngeal phase with slow and incomplete laryngeal rise, poor pharyngeal peristalsis, ineffective glottic protection, pooling in the piriform sinuses, or increasing the risk of penetration/aspiration [24]. Other EPS is the dystonic reaction, an acute muscle spasm affecting the face and neck, limiting the mobility of the mandible, tongue, pharynx, pharyngeal constrictor, and palatal elevation [24]. Finally, tardive dyskinesia due to prolonged use of antipsychotics is another EPS that produce choreo-athetoid movements (coordination of the face, jaw and tongue is affected). Tardive dyskinesia also generates dyskinetic movements of the pharynx and upper esophageal sphincter, delayed swallowing reflex and poor laryngeal elevation and closure [24]. Besides, the use of anticholinergics can produce xerostomia, hindering the creation and propulsion of the bolus [24]. They also produce sialorrhea, which increases the risk of aspiration [24]. Benzodiazepines produce sedation and inhibited gag, increasing the risk of choking and aspiration [24].

On the side of behavior, as an explanatory factor for risk of dysphagia, we find the behavioral manifestations typical of patients with SMI: tachyphagia or eating very fast, inadequate chewing (swallowing even without chewing), swallowing large boluses, PICA behaviors or keeping food in the mouth, which together with the negative symptoms of schizophrenia (inattention, abulia) can lead to choking [22, 24]. Kulkarni et al. [24], however, point to the lack of systematic studies that analyze the behavioral problems in these patients and their relation to dysphagia. In their study, these authors [24] indicate certain very negative consequences of the presence of dysphagia in SMI patients. Thus, the choking death rate is 85/100,000 in the psychiatric population, that is, 100% higher than in the general population [25], while the odds ratio for food-related choking death in patients with schizophrenia, as compared to the general population, is 7.92–12.80 (95% CI) [26].

The high prevalence and negative impact of these swallowing difficulties justify the effort to achieve early identification tools and intervention strategies for patients with cognitive impairment (CI) (dementia/ID) and well as for patients with SMI. Nonetheless, the very characteristics of these patients in regard to their cognitive and behavioral state (inattention, disorientation, memory problems, language difficulties in both comprehension and expression, introspection difficulties, unorganized thinking, episodes of mania/depression, difficulties in following instructions, etc.) often exclude the use of screening tests for risk of dysphagia, when these have been designed as self-reports, or their use has not been validated in these target populations [15, 22, 23, 27, 29–32]. In order to find a way around this drawback, there have been several attempts to develop screening methods that circumvent these difficulties. Thus, Michel et al. [27] carried out the first study to validate the Volume-Viscosity Swallow Test (V-VST) [28] in older adults with dementia. The V-VST is an OD screening test that assesses any alterations during swallowing, including clinical signs of impaired efficacy (labial seal, oral and pharyngeal residue, and piecemeal deglutition) and impaired safety of swallowing (voice changes, cough). The patient is tested through the intake of several types of food, of different bolus volume (5–20 ml) and viscosity (liquid, nectar, pudding). The results showed an 86.6% prevalence of swallowing disorder diagnosed by the V-VST (97 patients of the total 117), with an average test application time of 8.7 min. However, only 8.5% of the participants had severe CI in this study. The main obstacle in screening for OD with the V-VST is dealing with the patient’s cognitive state, which affects their ability to actively participate in the testing procedures. These patients may be lacking the skills in communication and following instructions that this procedure requires, especially patients with a higher degree of CI.

As an alternative to this assessment of dysphagia risk, part of the research team in the present study designed the Oropharyngeal Dysphagia Screening Test for Patients and Professionals (ODS-PP) [29]. The test consists of 18 four-point Likert items, organized into 3 assessment scales: Safety, Efficacy, and Other. The test was designed to be completed either by the patient with OD (self-reporting) or by the professional assigned to his/her food intake when there may be problems in the patient’s cognitive state. The first validation study of the ODS-PP included 206 older adults; 103 had a previous OD diagnosis, of which 55 also had cognitive impairment and whose test ratings were given by the professional assigned to their food intake. The instrument was able to significantly differentiate patients with previously diagnosed dysphagia from the control group participants, regardless of whether the dysphagia patients presented cognitive impairment. Thus, both groups of patients with OD (cognition preserved/cognition impaired) obtained significantly higher scores than the OD participants (control group) in all dimensions of the ODS-PP and in their total score, with a high effect size. Moreover, the ODS-PP showed adequate levels of reliability and construct and convergent validity (see Instruments under Method).

In the Netherlands, in relation to ID, the Dysphagia Disorders Survey (DDS) [30] has been used to screen for dysphagia. This 15-item questionnaire assesses dysphagia-related factors such as body mass index, diet consistency and body postural control, on one hand, and a meal-time assessment (feeding/swallowing competency including sensory motor components), reported by another person. The DDS is another report that must be completed by a certified speech therapist trained in the application of the instrument, thereby limiting its generalized use. Regarding the psychometric characteristics of the DDS, there are reports of moderately positive evidence for reliability (52–58%), content validity (64%), and structural validity (54%), and strong positive evidence for hypothesis testing (44–66%) in children with cerebral palsy and adults and children with ID [31]. Also in the Netherlands, Helder [quoted in 15] developed the Signaleringslijst Verslikken (SV), an 8-item questionnaire that screens for dysphagia risk. It is answered by professionals who deal directly with the adult ID patients but does not require previous training in its use. Helder found an interrater reliability of 90%, and a correlation of 0.7 between the SV and the DDS, with a 0.9 proportion of agreement between the two tests on either the presence or absence of dysphagia. In a later study, however, Timmeren et al. [15] concluded that there was insufficient convergent validity between the SV and the DDS for detecting risk of dysphagia in persons with profound ID (0.59), where the SV failed to identify 44% of the participants at risk for OD that were identified by the DDS. In this same country, the Screening Instrument for Dysphagia in ID (SD-ID) was recently developed as a quick and easy tool for daily caregivers to signal a risk for dysphagia [32]. In the validation study carried out with 1064 people with ID, the authors reported the SD-ID’s promising sensitivity and specificity (> 75%) compared to the DDS [32].

Finally, in the case of screening for dysphagia in SMI patients, the Hancox et al. review on nutrition risk screening methods for adults with SMI [22] only mentioned one article that performed a specific screening for dysphagia in these patients. This single study, by Regan et al. [23], applied a 9-item checklist on swallowing skills and symptoms of dysphagia in a self-report format to 60 patients with SMI. Even though the authors report that the checklist was able to detect risk of dysphagia in 19 of the 60 participants, the psychometric characteristics of the instrument were not established in the study.

Taking all this into consideration, the main aim of the present study was to go beyond the validation study of the Oropharyngeal Dysphagia Screening Test for Patients and Professionals (ODS-PP), by including in this case not only CI patients but also patients with SMI.

The specific aims are:

- To assess the validity of the ODS-PP test in screening for OD in patients with CI and in patients with SMI.
- To study the ODS-PP test’s psychometric feature of concurrent validity.
- To study the ODS-PP test’s psychometric feature of reliability / internal consistency.

It is hypothesized that the ODS-PP will be useful in screening for patients with possible oropharyngeal dysphagia without using food. In this way the safety of patients with cognitive deficits and patients with severe mental illness will not be compromised, because the professional responsible for the patient’s food intake will answer the questionnaire. The ODS-PP is expected to show appropriate psychometric qualities of concurrent validity and reliability.

## Method

### Participants

A total of 148 institutionalized patients (*M*_age_ = 72.4, *SD* = 9.37; 99 women and 49 men) participated in the study. Participants were divided into four subgroups: patients with cognitive impairment and OD (CI-OD, *n* = 28); patients with cognitive impairment and without OD (CI-n-OD, *n* = 50); patients with SMI and with OD (SMI-OD, *n* = 23); patients with SMI and without OD (SMI-n-OD, *n* = 47) (see participant characteristics in Table 1). All previous diagnoses of OD were made by neurologists, otorhinolaryngologists and/or speech therapists. Functional assessment protocols for dysphagia risk (observation of cough before/during/after meals, choking episodes, dehydration, nutrition, drooling, myofunctional evaluation, V-VST) and/or objective tests (laryngoscopy, video-fluoroscopy), given previous occurrence of aspiration pneumonia had been informed in the patient medical history. All participants diagnosed with OD had an adapted feeding (crushed diet). Participants without OD had a normal feeding. All participants were native Spanish speakers. All of them were of Caucasian origin except for one participant of Arab origin and another of African American origin.

**Table 1:** Participants’ sociodemographic information by group

			CI-OD	CI-n-OD	SMI-OD	SMI-n-OD
		*n*	28	50	23	47
		Mean age (*SD*)	74.93 (9.27)	75 (9,3)	75.83 (8.73)	66.59 (7.08)
		Mini Mental *(SD)*	4.15 (6.70)	10.7 (6.08)	16.7 (8.42)	26.51 (3.36)
	Sex	Male	8	10	12	19
		Female	20	40	11	28
Diagnoses	CI	Dementia: Alzheimer disease, frontotemporal dementia, Lewy body dementia or vascular dementia	17	27		
		Intellectual disability: moderate, severe, and profound	11	23		
	SMI	Schizophrenia and other psychotic disorders			21	44
		Bipolar disorder			1	1
		Major depressive disorder			1	2
		Length of time in institution (mean of years by group) (*SD*)	21.64 (22.88)	18.30 (19.50)	14.04 (11.16)	14.68 (11.25)

Note. CI-OD = patients with cognitive impairment and OD; CI-n-OD = patients with cognitive impairment and without OD; SMI-OD = patients with severe mental illness and with OD; SMI-n-OD = patients with severe mental illness and without OD.

Inclusion criteria were: to be older adults aged 60–95 years, living in an institution, considering the increasing OD prevalence among this population with previous diagnoses of cognitive impairment or with SMI. Due to the small number of users who met the inclusion criteria, especially in the OD subgroups, and considering symptomatic variability, it was not possible to apply the analyzes in each diagnostic category separately; therefore it was decided to make groups following the cognitive impairment category (dementia /intellectual disability) and SMI. In the case of dementia, users had different types of diagnoses (Alzheimer disease, frontotemporal dementia, Lewy body dementia or vascular dementia); the same happened in the case of ID, where the severity of the illness varied from moderate to severe, and profound. Likewise, in the case of the SMI category, the diagnostic and symptomatic variability of the patients prevented us from doing analysis by specific groups of disorders, therefore grouping under the SMI category patients with schizophrenia and other psychotic disorders (mostly), bipolar disorder, or major depressive disorder (according to DSM-5 or CIE-11 criteria).

As exclusion criteria, the study excluded the participation of patients with an adapted preventive diet (18 patients), whose swallowing was preserved, or they showed some kind of refusal to feed, due to behavioral disturbance (2 patients). Cases with doubtful diagnosis of dysphagia were also excluded.

Information about inclusion and exclusion criteria, as well as the other features for the sample selection, were collected from the clinic and caregiver professionals in the institution where the participants live.

### Instruments

*Mini Mental State Examination* (MMSE) [33] is a brief 11-question test that assesses mental status through five areas of cognitive function: orientation, registration, attention and calculation, recall, and language. The MMSE has been adapted to Spanish by Lobo et al. [34, 35] and it is the most commonly used test for standardized cognitive assessment in the clinical setting, especially in the case of older adults. The maximum score is 30 and a score of 23 or lower is indicative of cognitive impairment. The test takes around 5–10 min to administer. The MMSE’s inter-observer reliability is high with a mean kappa value of 0.97; internal consistency was represented by Cronbach’s alpha at the level of 0.75–0.78.

*The Oropharyngeal Dysphagia Screening Test for Patients and Professionals* (ODS-PP) [29]. The test consists of 18 four-point Likert-type items, organized in 3 assessment scales: Safety, Efficacy, and Other. In most of the items, the Likert scale ranged from 1 = Never to 4 = Very often. Some of the items had to be adapted to the Likert rating scale; for example, item 9 in the Efficacy scale, “In the last 6 months, how much weight have you/has (s)he lost? (0 kg, 1–5 kg, 5–10 kg, > 10 kg)” was rated in the Likert format as follows: 1 = 0 kg; 2 = 1–5 kg; 3 = 5–10 kg; 4 = > 10 kg. Another example, item 18, in the Other scale, “How long does eating take you/him/her?” was rated as follows: 1 = < 10′, 2 = 10–20′, 3 = 20–30′, 4 = > 30′. The test has been designed to be completed either by the patient with OD (self-reporting) or the professional taking care of his/her food intake when there may be problems in the patient’s cognitive state. The test score ranges from 18 (minimum) to 72 (maximum) points. In the first study with the ODS-PP [29], Cronbach’s alpha was 0.95 for the whole set of items and all participants; the Corrected Item-Total Correlation ranged from 0.51 to 0.83; Cronbach’s alpha if Item is Deleted ranged from 0.94 to 0.95. The reliability analysis of each scale also showed high results: Safety (8 items) α = 0.92, Corrected Item-Total Correlation from 0.65 to 0.84; Cronbach’s alpha if Item is Deleted from 0.89 to 0.91; Efficacy (5 items) α = 0.84, Corrected Item-Total Correlation from 0.53 to 0.72; Cronbach’s alpha if Item is Deleted from 0.79 to 0.84; and Other (5 items) α = 0.85, Corrected Item-Total Correlation from 0.47 to 0.80; Cronbach’s alpha if Item is Deleted from 0.78 to 0.87, the latter for item number 18 (How long does eating take you?). Reliability results excluding each of the test scales remained between 0.91 and 0.94. Correlation analysis of each single item and the total score for the ODS-PP test showed significant correlations in all cases with *r* values ranging between 0.54 and 0.86 (*p* = 0.001). Regarding concurrent validity, initial study results showed significant correlations of all items of the ODS-PP with items of the Eating Assessment Tool-10 (correlations ranged from *r* = 0.27 to *r* = 0.98, *p* = 0.001). Regarding the Swallowing Disturbance Questionnaire (SWAL-QOL), results showed significant correlations of all items of the ODS-PP test scales with the items of the SWAL-QOL test scales: Burden, Eating duration, Eating desire, Symptom Frequency, Food selection, Communication, Fear, Mental health, and Social (correlations ranged from − 0.18 to − 0.95; *p* ≤ 0.005, *p* ≤ 0.001). Partially significant correlations were found between all items of the ODS-PP test scales and the items from the SWAL-QOL test’s Fatigue dimension. Significant correlations between the items of the ODS-PP and the items from the SWAL-QOL Sleep dimension were nearly absent.

*The Eating Assessment Tool*-*10* (EAT-10) [36, 37] is a unidimensional screening test for the early detection of swallowing difficulties and dysphagia. It is a rapid test, which can be completed in about 2 min. It is composed of 10 Likert-type items and provides a raw score of 40 points (maximum) to 0 points (minimum); a high raw score is often seen as an indicator of dysphagia. There is a Spanish version, translated and adapted but without normative scores or specific cut-off points. The reliability of this version is a 0.878 Cronbach alpha, the mean patient score is 15 points, and dysphagia risk is 6.7. It is the only OD screening test validated in Spain, which is why it is selected as an instrument to determine concurrent validity.

*The Swallowing Disturbance Questionnaire* (SWAL-QOL) [38–41] is a test for the assessment of the impact of OD on quality of life (QOL). It consists of the following 11 multi-item scales: Burden, Eating duration, Eating desire, Symptom Frequency, Food selection, Communication, Fear, Mental Health, Social, Fatigue, and Sleep. It addresses recommendations about food, liquid, and dysphagia treatment and satisfaction with treatment. The questionnaire helps to identify patients with OD and is sensitive to differences in severity level using 44 Likert-style items, from 5—severe state to 1—least severe state. The score ranges from 44 (worst QOL) to 220 (best QOL). There are no specific cut-off points but any score under 70 indicates problems in QOL. The original test’s reliability is a 0.82 Cronbach alpha. The Spanish adaptation process, employing a forward–backward–forward translation technique, is currently ongoing and no psychometric data are currently available [40]. Mean time of application for the Spanish version is about 21 min. Despite being an instrument to evaluate the impact of OD on quality of life, its use as a measure of concurrent validity is determined for several reasons. Firstly, due to the scarcity of translated/validated tests in the Spanish population. Secondly, because several of its subscales evaluate dimensions concurrent with the ODS-PP test, such as Eating duration, Eating desire, Symptom Frequency, Food selection, Communication.

### Procedure

Ethical approval for this study was obtained from the Ethics Committee of Clinical Research (CEIC-CEI) FIDMAG Germanes Hospitalàries..

The research was conducted at the Benito Menni Mental Health Care complex (SantBoi de Llobregat, Barcelona) . The University of Granada and the Board of the FIDMAG Germanes Hospitalàries Research Foundation signed a collaboration agreement to carry out the study. The selection of possible participants that match the inclusion/exclusion criteria was made by the Hospital Board, consisting of healthcare staff at the hospital. Due to patients’ impairment in cognitive state or mental health, the consent form was signed by their legal guardian after they received pertinent information about the study.

Seven professionals caregiver (nurses and nursing assistants) took part in the research. They all signed a consent form. They were instructed in the ethical requirements of the study. Two formative meetings, with a total of three hours were developed by one of the authors, also the head nurse of the institution. In those meetings the research protocol and the assessment instruments were introduced. The head nurse checked the comprehension of items’ tests and the assessment procedure.

All the professionals taking care of the participants’ feeding had received specific instruction in courses on caring for patients with swallowing difficulties. Adapted diets catered for the patients were prescribed by the medical team and meals were prepared in the same care center. The above-mentioned professionals were responsible for preparing the beverages using thickeners and for feeding support. All the professionals who answered the tests had been instructed and had sufficient and broad knowledge about the population they were assisting; they knew about their way of eating, duration, usual type of food, and symptoms. All the nurses and nursing assistants were stable staff at the hospital unit with 36.25 h/week of working hours and a minimum of two years of contact with the participants of the study.

In the first session, the MMSE was used for the cognitive state assessment of all participants. In a second session (around 35 min), the professionals taking care of the participants’ feeding (nurses and nursing assistants) completed the ODS-PP test (around 10 min), the EAT-10 test (around 5 min), and the SWAL-QOL tests (around 20 min) following a counterbalanced order.

### Design and Data Analysis

Analyses were performed using the Statistical Package for the Social Sciences (SPSS), version 29.0. The study followed an ex post facto design. Independent variable (by selection) was Group, with four levels: CI-OD, CI-n-OD, SMI-OD and SMI-n-OD. Multivariate Analysis of variance (MANOVA) was used to assess the differences in order to determine screening validity; differences were presented as percentages and a 95% confidence interval. Pearson’s correlation coefficients were calculated for determining the concurrent validity between the ODS-PP and the EAT-10 and the SWAL-QOL. Cronbach’s alpha and Pearson’s correlation coefficient were calculated in order to study the correlations and the test reliability value—internal consistency. Univariate Analysis of Variance (ANOVA) was calculated to analyze any possible age differences and the Chi-square test was used to compare qualitative values (Sex).

## Results

There were significant differences between group participants as a function of Sex, *χ*^2^(3) = 9.051, *p* = 0.03 and also as a function of Age *F*(3,144) = 10.793, *p* = 0.0001, specifically between SMI-n-OD and the other three groups (*p* = 0.0001 in all cases). There were not significant differences between groups as function of Years in the Institution *F*(3,144) = 1.323, *p* = 0.26.

**Fig. 1:**
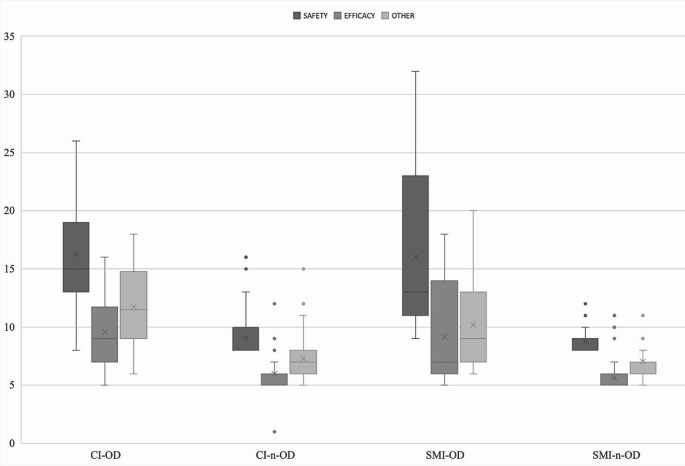
Distribution of ODS-PP subscale scores by subgroup Note. CI-OD = patients with cognitive impairment and OD; CI-n-OD = patients with cognitive impairment and without OD; SMI-OD = patients with severe mental illness and with OD; SMI-n-OD = patients with severe mental illness and without OD

*Study of the ODS-PP test for screening OD in patients with CI or SMI*. In order to test the first objective, that is, to assess the ODS-PP test’s screening validity for differentiating between participants with and without OD in patients with CI or SMI, a MANOVA was carried out to compare the 4 groups CI-OD, CI-n-OD, SMI-OD and SMI-n-OD. Results showed significant differences among the groups when considering the whole test scores *λ* = 0.461, *F*(12,373) = 10.566, *p* = 0.0001, *η*^2^ = 0.23, *sp*. = 1; there were also significant differences considering each scale: Safety, Efficacy, Other, and Total ODS-PP score (see Table 2 and Fig. 1). Post hoc analysis (Bonferroni) showed significant differences among CI-OD and the other two groups without OD (CI-n-OD and SMI-n-OD) in Safety, Efficacy, Other, and Total score. The same pattern was found between the SMI-OD and the two groups without OD (CI-n-OD and SMI-n-OD). There were no significant differences between the two groups with OD (CI-OD and SMI-OD) nor between the two groups without OD (CI-n-OD and SMI-n-OD). Post hoc results are shown in Table 3.

**Table 2:** MANOVA results in each of the ODS-PP test scales

				M	SD	F(3,144)	*p*	η^2^	Statistic Power	
Safety	CI-OD		16.28		4.70	44.312	0.0001	0.48		1
	CI-n-OD		9.04		1.89					
	SMI-OD		16.00		6.91					
	SMI-n-OD		8.77		1.32					
Efficacy	CI-OD		9.61		3.13	22.084	0.0001	0.31		1
	CI-n-OD		6.00		1.74					
	SMI-OD		9.17		4.59					
	SMI-n-OD		5.64		1.29					
Other	CI-OD		11.71		3.42	29.008	0.0001	0.37		1
	CI-n-OD		7.30		1.84					
	SMI-OD		10.17		3.95					
	SMI-n-OD		7.02		1.03					
Total	CI-OD		37.53		8.77	48.473	0.0001	0.50		1
	CI-n-OD		22.32		3.90					
	SMI-OD		35.39		13.77					
	SMI-n-OD		21.04		2.24					

Note. CI-OD = patients with cognitive impairment and OD; CI-n-OD = patients with cognitive impairment and without OD; SMI-OD = patients with severe mental illness and with OD; SMI-n-OD = patients with severe mental illness and without OD.

**Table 3:** Post hoc comparisons between subgroups in each of the ODS-PP test scales

	Bonferroni	*p*	d	r
Safety	CI-OD vs. CI-n-OD	0.0001	2.02	0.71
	CI-OD vs. SMI-n-OD	0.0001	2.17	0.74
	SMI-OD vs. CI-n-OD	0.0001	1.37	0.57
	SMI-OD vs. SMI-n-OD	0.0001	1.45	0.59
	CI-OD vs. SMI-OD	1	0.047	0.02
	CI-n-OD vs. SMI-n-OD	1	0.16	0.08
Efficacy	CI-OD vs. CI-n-OD	0.0001	1.42	0.58
	CI-OD vs. SMI-n-OD	0.0001	1.66	0.64
	SMI-OD vs. CI-n-OD	0.0001	0.92	0.42
	SMI-OD vs. SMI-n-OD	0.0001	1.05	0.47
	CI-OD vs. SMI-OD	1	0.11	0.05
	CI-n-OD vs. SMI -n-OD	1	0.23	0.11
Other	CI-OD vs. CI-n-OD	0.0001	1.60	0.63
	CI-OD vs. SMI-n-OD	0.0001	1.86	0.68
	SMI-OD vs. CI-n-OD	0.0001	0.93	0.42
	SMI-OD vs. SMI-n-OD	0.0001	1.86	0.68
	CI-OD vs. SMI-OD	0.167	0.41	0.20
	CI-n-OD vs. SMI -n-OD	1	0.18	0.09
Total	CI-OD vs. CI-n-OD	0.0001	2.24	0.75
	CI-OD vs. SMI-n-OD	0.0001	2.52	0.78
	SMI-OD vs. CI-n-OD	0.0001	1.29	0.54
	SMI-OD vs. SMI-n-OD	0.0001	1.42	0.58
	CI-OD vs. SMI-OD	1	0.18	0.09
	CI-n-OD vs. SMI -n-OD	1	0.40	0.19

Note. CI-OD = patients with cognitive impairment and OD; CI-n-OD = patients with cognitive impairment and without OD; SMI-OD = patients with severe mental illness and with OD; SMI-n-OD = patients with severe mental illness and without OD.

*Study of the ODS-PP test’s psychometric feature of concurrent validity.* Correlation analyses were carried out to analyze concurrent validity of the ODS-PP test compared to the EAT-10 test on one part, and the SWAL-QOL test on the other, with these specific populations. Results showed significant correlations of all scales of the ODS-PP with the EAT-10 test total score: Safety (*r* = 0.89, *p* = 0.001); Efficacy (*r* = 0.77, *p* = 0.001); Other (*r* = 0.73, *p* = 0.001); and Total ODS-PP score (*r* = 0.92, *p* = 0.001).

Regarding the relationship between the ODS-PP and the SWAL-QOL tests, results showed significant correlations of all the ODS-PP test scales with the SWAL-QOL test’ scales: Burden, Eating duration, Eating desire, Symptom Frequency, Food selection, Communication, Fear, Mental health, Social, Fatigue and Sleep (correlations ranged from − 0.18 to − 0.90 (*p* ≤ 0.005, *p* ≤ 0.001). The correlation matrix is shown in Table 4.

**Table 4:** Correlation matrix of the ODS-PP test scales and the SWAL-QOL test scales

	ODS-PP Safety	ODS-PP Efficacy	ODS-PP Others	ODS-PP Total
Burden	-0.71**	-0.73**	-0.39**	-0.70**
Eating duration	-0.66**	-0.68**	-0.52**	-0.70**
Eating desire	-0.63**	-0.61**	-0.44**	-0.64**
Symptom Frequency	-0.89**	-0.85**	-0.61**	-0.90**
Food selection	-0.63**	-0.58**	-0.28**	-0.58**
Communication	-0.64**	-0.79**	-0.45**	-0.71**
Fear	-0.64**	-0.68**	-0.30**	-0.63**
Mental health	-0.65**	-0.68**	-0.34**	-0.64**
Social	-0.71**	-0.77**	-0.41**	-0.72**
Fatigue	-0.61**	-0.63**	-0.41**	-0.63**
Sleep	-0.26**	-0.23**	-0.18*	-0.26**
Total	-0.83**	-0.85**	-0.53**	-0.85**

** *p* = 0.001

*Study of the ODS-PP Test’s psychometric feature reliability—internal consistency.* Cronbach’s alpha (*α*) coefficient was computed to estimate the ODS-PP test’s reliability—internal consistency. Results showed that the ODS-PP test was highly reliable (Cronbach’s alpha, *α* = 0.94, for the whole set of items and all participants; Corrected Item-Total Correlation 0.07 (item 18 *How long does eating take you?*) and rest of items from 0.54 to 0.85; Cronbach’s alpha if Item is Deleted, from 0.93 to 0.94). Reliability analysis of each scale also showed high results: Safety (8 items) *α* = 0.92, Corrected Item-Total Correlation from 0.67 to 0.83; Cronbach’s alpha if Item is Deleted from 0.91 to 0.93; Efficacy (5 items) *α* = 0.85, Corrected Item-Total Correlation from 0.50 to 0.81; Cronbach’s alpha if Item is Deleted from 0.77 to 0.86; and Other (5 items) *α* = 0.80, Corrected Item-Total Correlation 0.25 (item 18) and rest of items from 0.60 to 0.76; Cronbach’s alpha if Item is Deleted from 0.70 to 0.85. Reliability results excluding each of the test scales remained among 0.66 and 0.83.

Correlation analysis of each single item and the total score for the ODS-PP test showed significant correlations in all cases, except for item 18 (see Table 5), with *r* values ranging between 0.54 and 0.86 (*p* = 0.001), thus supporting the suitability of the test items.

**Table 5:** Correlation matrix of each ODS-PP test´s item (1–18) and the ODS-PP total score

	1	2	3	4	5	6	7	8	9	10	11	12	13	14	15	16	17	18
OD-PP total	0.80**	0.79**	0.83**	0.80**	0.87**	0.77**	0.75**	0.74**	0.61**	0.59**	0.82**	0.79**	0.68**	0.71**	0.59**	0.82**	0.76**	0.14

** *p* = 0.001

Regarding the analysis of groups with OD, results showed Cronbach’s alpha = 0.88 for the whole set of items in CI-OD. Reliability analysis of each scale showed Safety (8 items) *α* = 0.88, Efficacy (5 items) *α* = 0.81, and Other (5 items) *α* = 0.78. Reliability results excluding each of the test scales remained between 0.16 and 0.77. Regarding SMI-OD, results showed Cronbach’s alpha = 0.95, for the whole set of items. Reliability analysis of each scale showed Safety (8 items) *α* = 0.94, Efficacy (5 items) *α* = 0.93, and Other (5 items) *α* = 0.86. Reliability results excluding each of the test scales remained between 0.66 and 0.85.

## Discussion

This study aimed to support the validity of the Oropharyngeal Dysphagia Screening Test for Patients and Professionals (ODS-PP test) as a systematic screening for OD, not only in the general population of older adults, but also in populations with severe impairments, thereby extending its validation, in each case, for populations with dementia and intellectual disability, and also for patients with severe mental illness.

The results of this study show adequate diagnostic validity of the ODS-PP when differentiating participants previously diagnosed with OD from participants that did not present OD, regardless of the primary disease (dementia/ID or SMI), with high effect sizes. Bear in mind that the ODS-PP was administered to the professionals who were responsible for the participants’ food intake. In this regard, the results follow in the line of the previous study [29]. The ODS-PP has also shown adequate convergent validity and reliability levels even higher than those of the initial test study in the OD subgroups. Specifically, in the first study with the ODS-PP, Cronbach’s alpha for the whole set of items was *α* = 0.53 in the preserved-cognition OD subgroup (the participants answered the ODS-PP) and *α* = 0.69 in the altered-cognition OD subgroup (the professionals responsible for their food intake answered the ODS-PP). In the present study, Cronbach’s alpha for the whole set of items was *α* = 0.88 in the CI-OD subgroup and *α* = 0.95 in the SMI-OD subgroup. The same results are observed with the subscales (preserved-cognition OD subgroup): Safety *α* = 0.54, Efficacy *α* = 0.48, and Other *α* = 0.34; altered-cognition OD subgroup: Safety *α* = 0.63, Efficacy *α* = 0.62, and Other *α* = 0.74; CI-OD subgroup: Safety *α* = 0.88, Efficacy *α* = 0.81, and Other *α* = 0.78; SMI-OD subgroup: Safety *α* = 0.94, Efficacy *α* = 0.93, and Other *α* = 0.86. The data from this second study also concur with the first study in problems related to item 18 of the Other subscale (How long does eating take you?); inclusion of this item in future applications must be reconsidered. Consequently, these results follow the line of the preliminary study of ODS-PP [29] and increase the robustness of these conclusions.

Although there seems to be a trend towards the design and validation of OD screening tests in populations with CI (dementia/ID) [15, 27, 29, 30, 32], the instruments available so far present some practical difficulties. For example, although the study by Michel et al. [27] concludes that V-VST is valid for screening older adults with dementia, only 8.5% of the participants in this study presented severe CI. Even though these results are promising, the V-VST requires the patient to present an alert state, follow instructions and understand the language when the test is applied --requirements that may not be met in patients with severe CI. Both the V-VST and the DDS [30] require that professionals responsible for test administration have received training and/or initial certification, thus limiting their widespread use. For its part, the SD-ID [32] shows promising initial results, although the population where it has been validated is limited to people with ID. The ODS-PP [29], however, has proven to be reliable and valid both in older adults without primary diseases, as well as in patients with dementia, with ID, and even more groundbreaking, in persons with SMI. As Hancox et al. [22] underscored in their review, only one study [23] had initiated a screening procedure for dysphagia in a population with SMI, but without validating the instrument. Therefore, the present study extends the validation data of the ODS-PP, a brief instrument with simple language. It can be used either as a self-report or in its other-report format in older adults that present risk for dysphagia, whether their cognitive state is preserved or altered, in addition to its use in SMI patients. Since it does not require the use of food, assessment safety is increased. Moreover, it does not require previous training or certification of the healthcare professionals prior to its administration.

The present study has overcome some of the limitations of the preliminary study [29]. For example, the geographic area of the sample was extended (assessments in the preliminary study were conducted in the south of Spain; in this case we assess a population in the northwest). Subgroups with specific primary pathologies were identified: dementia/ID and SMI. We included a new control group to assess patients with CI but not with OD. However, despite the efforts made, the present study also has a number of limitations. First, we mention the small sample size and its local nature. This motive prevented us from working separately with the dementia and ID subgroups, or from differentiating by type of dementia or severity of ID. Similarly, we could not differentiate between the different disorders that make up the subgroups with SMI. Another limitation refers to the use of the EAT-10 and the SWAL-QOL as measures of convergent validity. However, there is no other test in Spain to date, aside from the ODS-PP, that has been validated with professionals responsible for patients’ food intake. It is therefore necessary to continue working on the validation of the ODS-PP, expanding its target groups, analyzing its validity according to type of dementia, severity of ID, including new primary pathologies such as Parkinson’s, Multiple Sclerosis or myasthenia gravis, Amyotrophic Lateral Sclerosis, stroke and cancer of the pharynx or larynx. In addition, in view of the few OD-screening studies that have been conducted to date in patients with SMI, it would be advisable to increase the population of patients with schizophrenia and other psychotic disorders, bipolar disorder, or major depressive disorder, for the purpose of doing specific analyses by primary pathology. Finally, it would be very interesting to validate the use of the ODS-PP with family members or informal caregivers, as has been done by Schüller-Korevaar et al. [32] with the SD-ID; otherwise, application of the ODS-PP would be limited to institutionalized patients.

In conclusion, the current study further establishes the ODS-PP as a valid screening test for the detection of OD in older adults with CI or SMI. Our main aim was to provide healthcare communities with a valid, easy-to-use tool that can be applied without the patient’s active participation and without compromising their safety, and that could be included in the diagnostic protocols for evaluating the risk of OD. With this tool, the formal diagnosis of dysphagia in these populations would increase (traditionally underdiagnosed) and consequently, the use of measurement and intervention strategies would improve, assuring safety and efficacy in the feeding conditions of these patients.

## Data Availability

This research data cannot be shared openly, in order to protect study participants’ individual privacy. Appropriate restrictions before sharing their data should be applied and if required, each participant consent should be obtained and documented prior to data sharing.
